# Wide Distribution of Virulence Genes among *Enterococcus faecium* and *Enterococcus faecalis* Clinical Isolates

**DOI:** 10.1155/2014/623174

**Published:** 2014-07-15

**Authors:** Sara Soheili, Sobhan Ghafourian, Zamberi Sekawi, Vasanthakumari Neela, Nourkhoda Sadeghifard, Ramliza Ramli, Rukman Awang Hamat

**Affiliations:** ^1^Department of Medical Microbiology and Parasitology, Faculty of Medicine and Health Sciences, Universiti Putra Malaysia, 43400 Serdang, Malaysia; ^2^Clinical Microbiology Research Center, Ilam University of Medical Sciences, Ilam, Iran; ^3^Department of Medical Microbiology and Immunology, Medical Faculty, Universiti Kebangsaan Malaysia Medical Center, Cheras, 56000 Kuala Lumpur, Malaysia

## Abstract

*Enterococcus*, a Gram-positive facultative anaerobic cocci belonging to the lactic acid bacteria of the phylum Firmicutes, is known to be able to resist a wide range of hostile conditions such as different pH levels, high concentration of NaCl (6.5%), and the extended temperatures between 5^°^C and 65^°^C. Despite being the third most common nosocomial pathogen, our understanding on its virulence factors is still poorly understood. The current study was aimed to determine the prevalence of different virulence genes in *Enterococcus faecalis* and *Enterococcus faecium*. For this purpose, 79 clinical isolates of Malaysian enterococci were evaluated for the presence of virulence genes. *pilB, fms8, efaAfm*, and *sgrA* genes are prevalent in all clinical isolates. In conclusion, the pathogenicity of *E. faecalis* and *E. faecium* could be associated with different virulence factors and these genes are widely distributed among the enterococcal species.

## 1. Introduction

Enterococci are considered to be part of normal gut microbiota in both humans and animals and capable to survive in a diverse range of harsh conditions. Among them only two, that is,* Enterococcus  faecium* and* Enterococcus  faecalis,* are now being increasingly recognized to be involved in human infections such as bacteremia, endocarditis, urinary tract infections, and surgical site infections [1, 2]. This could be explained by their inherent resistance to various antibiotics with greater adaptability in hospital environments and has high genetic diversity as well as the presence of various virulence factors [3–5].

These opportunistic bacteria possess various virulence factors including enterococcal surface protein (Esp) and aggregation substance (Agg) which could enhance the colonization process in the host and binding to the host epithelium, respectively [6]. Others such as cytolysin [7], enterolysin A [8], gelatinase [9], hyaluronidase, Zn-metalloendopeptidase, enhanced expression of pheromone (Eep) [10], and adhesion-associated protein EfaA (*E. faecalis* endocarditis antigen A) [11] have been reported to be among the most important virulence factors.

Surprisingly through NCBI database analysis, there are some similarities observed in terms of their virulence determinants among enterococci. For instance, pilA in* E. faecium* has 99% similarity with the cell wall surface anchor family protein of* Enterococcus* sp. GMD4E (Accession: EKA01662). The pilB of* E. faecium* has 74% similarity with the cell wall surface anchor protein of* E. faecalis* B318 (Accession: ETU21897). Meanwhile, the ecbA in* E. faecium* has 92% similarity with the cell wall surface anchor protein of* E. faecalis* (Accession: WP_010708790) and 91% with Cna protein B-type domain protein* E. faecalis* (Accession: WP_002417581) (these regions are part of a collagen binding MSCRAMM, ecbA).

The cell wall adhesion (efaAfm) in* E. faecium* has 66% similarity with endocarditis specific antigen of* E. faecalis* T8 (Accession: EEU25576). With regard to the collagen binding MSCRAMM Acm (fms8) in* E. faecium*, 57% similarity has been observed with the collagen binding domain protein of* E. faecalis* (Accession: WP_016632409). The blast analysis for the collagen binding MSCRAMM scm in* E. faecium* has revealed 99% similarity with the collagen-binding MSCRAMM scm (Fms10) in* Enterococcus* sp. GMD5E. Finally, 37% similarity of the cell wall anchored protein sgrA in* E. faecium* has been detected when compared to the surface adhesion protein in* Enterococcus* sp. C1 (Accession: WP_008378446). These findings could be related to the notion that horizontal transfer of both resistance and virulence determinants is very common among enterococci [12]. Thus, this study was conducted to investigate the distribution of the diverse virulence factors and specify the dominant virulence genes among* E. faecalis* and* E. faecium* clinical isolates. Based on the similarity results and also the possibility of horizontal virulence gene transfer the* pilA, pilB, hyl, ecbA, scm, fms8, efaAfm,* and* sgrA* genes were chosen for this study.

## 2. Material and Methods

### 2.1. Bacterial Isolates

A total of 79 clinical isolates of* E. faecalis *(50 isolates) and* E. faecium* (29 isolates) were identified during May 2009 to March 2010 from a tertiary teaching hospital. Nonrepetitive isolates were collected from these samples such as urine, blood, pus, vaginal, and sterile body fluid.

### 2.2. DNA Extraction of* Enterococcus*

The DNA wasextracted using the DNA extraction kit (Gene ALL, South Korea) according to the manufacturer's instructions.

### 2.3. Evaluation of Different Virulence Genes

All isolates were subjected to the amplified* pilA, pilB, hyl, ecbA, scm, fms8, efaAfm,* and* sgrA* genes, using specific primers as listed in Table 1. These virulence genes are most strongly associated with clinical lineages of* E. faecium*; however, not much data has been reported among* E. faecalis.* The PCR amplification was carried out in a DNA thermocycler (Bio-Rad) using the amplification parameters as follows: initial denaturation at 95°C for 2 minutes, followed by 35 cycles of denaturation at 95°C for 20 seconds, annealing at 58°C for 10 seconds, and extension at 72°C for 20 seconds, with a final extension at 72°C for 5 minutes. All PCR products were analyzed by 1% agarose gel electrophoresis.

### 2.4. Purification of Virulence Genes from Agarose Gel

PCR products of each virulence gene were run on 1% (w/v) molecular grade agarose gel (Sigma, USA), using a Bio-Rad mini-gel electrophoresis system at 80 V for 70 minutes. The DNA was purified using DNA purification kit (Gene ALL, South Korea) according to the manufacturer's instructions.

### 2.5. Sequence Analysis

The PCR products were purified from gel agarose and then the purified products were sequenced by Sigma Company (Singapore). The results of DNA sequencing were run in Chromas Lite program to analyze the similarity to the sequenced gene in GenBank library.

## 3. Results and Discussion

**Figure 1:**
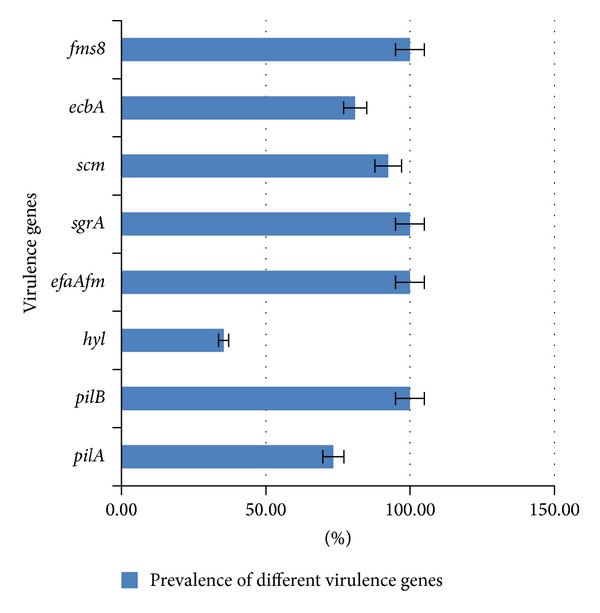
Prevalence of different virulence genes in clinical* E. faecium* and* E. faecalis *isolates. The* pilB,  fms8,  efaAfm,* and * sgrA *were identified in all clinical isolates.

A total of 79* E. faecalis *(50 isolates) and* E. faecium *(29 isolates) were analyzed for the presence of different virulence genes including* pilA, pilB, hyl, ecbA, scm, fms8, efaAfm, *and* sgrA*. The analysis showed different prevalence of virulence genes in* Enterococcus *which ranged from 35.4% to 100%. The* pilB, fms8, efaAfm,* and* sgrA* were identified as the dominant virulence genes in all isolates. The second most prevalent virulence gene,* scm*, was found in 92.4% of the isolates (*n* = 73/79). The* ecbA *was determined as the third most common prevalent virulence gene with 81% (*n* = 64/79) frequency. The* pilA* showed 73.4% (*n* = 58/79) prevalence in clinical isolates of* Enterococcus*. The lowest prevalence detected was* hyl* with the prevalence of 35.4% (*n* = 28/79). The prevalence of different virulence genes is shown in Figure 1. With regard to both enterococcal species, the analysis showed that all selected virulence genes were positive among* E. faecium *isolates. The exception is* hyl *where it was detected with 82.7% prevalence rate. Among* E. faecalis* isolates,* pilB, fms8, efaAfm*, and* sgrA* genes were detected in all isolates, followed by* scm* (88%),* ecbA* (70%), and* pilA* (58%). The least prevalence was* hyl* which was detected in only 4 isolates (8%).  Table 2 shows the distribution of different virulent determinants among* E. faecium* and* E. faecalis* clinical isolates. We believe that this is the first report on the prevalence of selected virulence genes among* E. faecalis* which could be demonstrated by the possibility of horizontal gene transfer among* E. faecium *and* E. faecalis.*

Pili which are also known as fimbriae have been detected in Gram-positive bacteria [13–15]. This surface organelle is responsible for endocarditis and biofilm formation in Gram-positive bacteria [16], and mediates attachment to human epithelium and skin [17] and confers resistance against macrophages [18].

**Figure 2:**
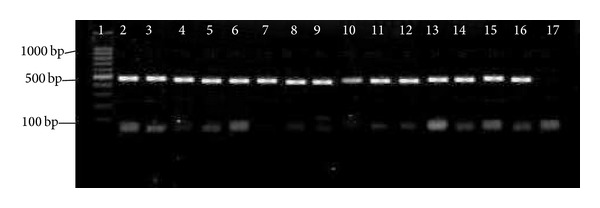
PCR results of * pilA*. 1 = marker = 100 bp; 2–16 =* pilA* = 495 bp; 17 = negative control.

**Figure 3:**
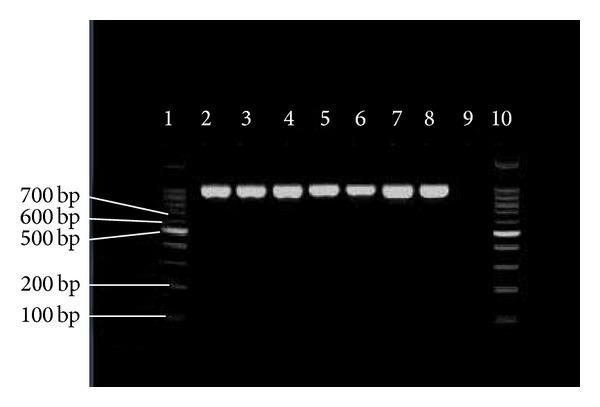
PCR results of* pilB*. 1, 10 = marker = 100 bp; 2–8 =* pilB* = 959 bp; 9 = negative control.

So far, limited data has been documented on the prevalence of pili in* E. faecium*. In our study, high prevalence rate of* pilB* in clinical* E. faecium *and* E. faecalis* isolates (100% for each) has been observed. In comparison with* pilB*, 21 (26.6%) clinical* E. faecalis* isolates were negative for the presence of* pilA*. Figures 2 and 3 show the presence of* pilA* and* pilB* genes in representative isolates. Hendrickx et al. [19] have demonstrated the presence of* pilA* and* pilB* in clinical isolates of* E. faecium.* Their analysis showed that* pilA* could not be expressed in broth condition and the best temperature for their expression was 37°C. This could probably be a reason as to why we could not detect* pilA* in some of our clinical* E. faecalis* isolates. Another possible reason could be explained by the lack of horizontal gene transfer among* E. faecalis *isolates.

Nowadays, it is obvious that in pathogenic bacteria several proteins have evolved to adhere to and invade into the host cells and subsequently escape and resist host defense [20]. MSCRAMMs which are known as microbial surface components recognizing adhesive matrix molecules have an important role for cell adhesion and involve in pathogenicity of bacteria [21].* In silico* analysis has revealed a family of genes that encode MSCRAMM-like proteins in* Enterococcus* such as* ebp *(endocarditis and biofilm-associated pilus of* E. faecalis*) operon,* ace* (adhesion of collagen from* E. faecalis*), and* acm* (adhesion of collagen from* E. faecium*) [22–24].

**Figure 4:**
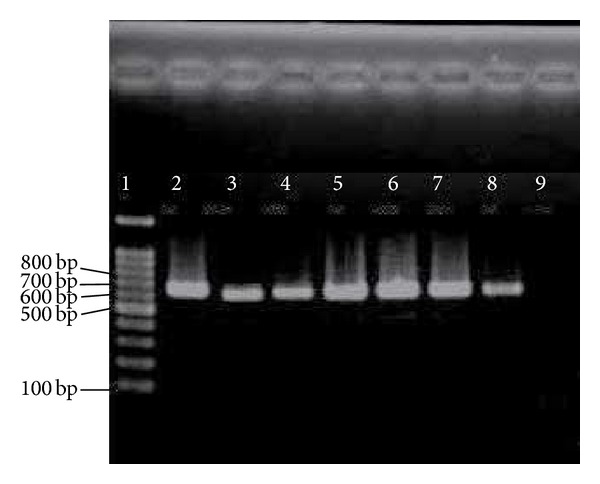
PCR results of * fms8*. 1 = marker = 100 bp; 2–8 =* fms8* = 765 bp; 9 = negative control.

The role of collagen-binding MSCRAMM* acm *(*Fms8*) is to bind to the collagen types I and IV. These types of collagen are also an important antigen involved in human during endocarditis. In the current research, high prevalence rate of* fms8* (100% for each) was detected among* E. faecium* and* E. faecalis* clinical isolates and could play an important role in pathogenicity of both enterococcal species.  Figure 4 shows the presence of* fms8* genes in representative isolates.

**Figure 5:**
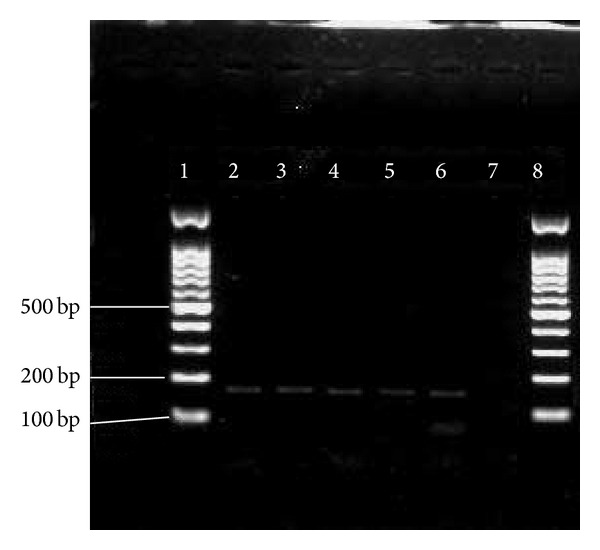
PCR results of* efaAfm*.  1, 8 = marker = 100 bp;  2–6 =* efaAfm* = 199 bp; 7 = negative control.

The exact role of* efaAfm* is unknown although it is believed to be involved in cell wall adherence. The* efaAfm* gene was only found in* E. faecium* isolates [25]. A study by Barbosa et al. [26] has demonstrated 27% of  isolates harbored the* efaAfm* gene while the current study showed all isolates were positive for* efaAfm*. The presence of* efaAfm* gene among representative isolates is shown in Figure 5. Our finding corroborates with Martin et al. [27] where they have demonstrated all* E. faecium* isolates carrying* efaAfm *virulence gene.

**Figure 6:**
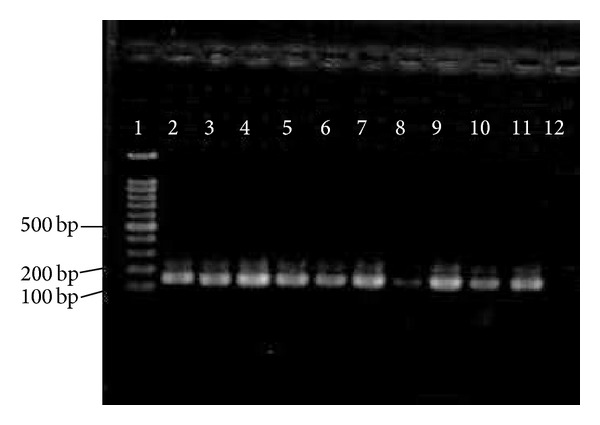
PCR results of* sgrA*. 1 = marker = 100 bp;  2–11 =* sgrA* = 150 bp;  12 = negative control.

**Figure 7:**
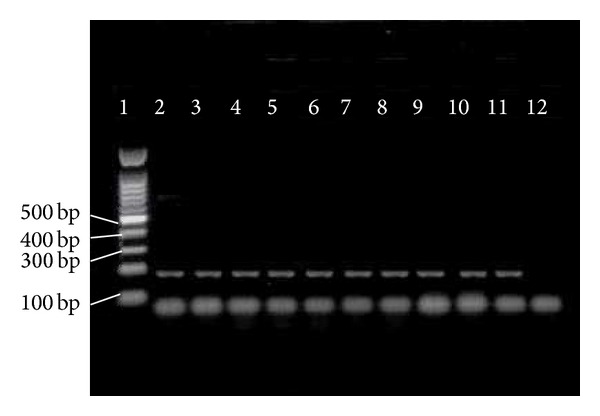
PCR results of* ecbA*. 1 = marker = 100 bp;  2–11 =* ecbA* = 182 bp;  12 = negative control.

The pathogenic* E. faecium* is enriched with two* orf2351* and* orf2430* genes which encode the* sgrA* and* ecbA *LPXTG-like cell wall anchored proteins, respectively.* sgrA* is a surface adhesion that can bind to the extracellular matrix molecules nidogen 1 and nidogen 2 and is also involved in biofilm formation. Meanwhile,* ecbA* binds to the collagen type V and fibrinogen. Both* ecbA *and* sgrA* were reported to be prevalent in clinical strains of* E. faecium* [28]. However, our study demonstrated that* sgrA *was more prevalent than* ecbA* (100% versus 81%). The representative isolates for the presence of* sgrA* and* ecbA* genes are shown in Figures 6 and 7.

**Figure 8:**
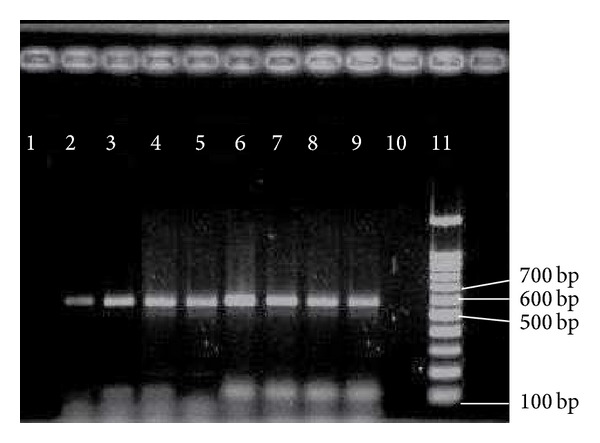
PCR results of * hyl*. 11 = marker = 100 bp;  2–9 =* hyl* = 605 bp; 1, 10 = negative control.

In addition,* hyl* virulence gene encodes for a putative glycosyl hydrolase that is considered as a plasmid harboring gene which has colonized the gastrointestinal tracts of mice subsequently caused an increase of pathogenicity of* E. faecium* in experimental peritonitis [29]. In a study by Panesso et al. [30] that subjected 32 hospitals in Colombia, Ecuador, Peru, and Venezuela, the results revealed that 23% of* E. faecium *strains carried the* hyl *virulence gene. Our results are in accordance with the previous study by Panesso et al. [30]. The* hyl* gene among representative isolates is shown in Figure 8. In a research by Worth et al. [31] among Australian haematology patients, only 2.1% of isolates showed positive reaction to* hyl*. A study by Vankerckhoven et al. [32] revealed 71% of VRE and 29% of vancomycin sensitive* Enterococcus* (VSE) harbored* hyl* virulence gene. Since* hyl* is not prevalent in all clinical isolates in our study, we believe that this gene could has little role in pathogenicity of* Enterococcus *in comparison with other prevalent virulence genes.

**Figure 9:**
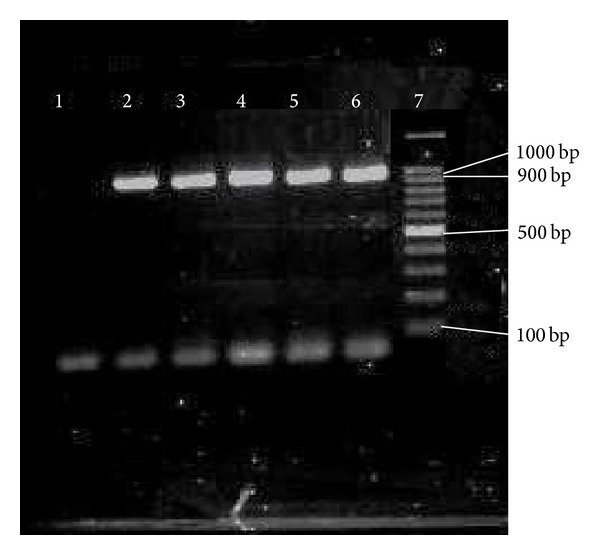
PCR results of* scm*. 7 = marker = 100 bp; 1–5 =* scm* = 1015 bp; 1 = negative control.

*Scm* (second collagen adhesion of* E. faecium*) binds to collagen type V and fibrinogen and it is commonly distributed among clinical and nonclinical isolates of* E. faecium*.* Scm* was first described in* E. faecium *in 2008 [33] but limited information on the prevalence of* scm *in* E. faecium *has been reported. Nonetheless, we documented a high prevalence of* scm* in clinical isolates of* E. faecium *(92.4%). Isolates representing for the presence of* scm* gene are shown in Figure 9.

## 4. Conclusion

The wide distribution of several virulence genes that is* pilB*,* fms8*,* efaAfm, *and* sgrA* in* E. faecalis* and* E. faecium* clinical isolates could give a clue that these virulence genes might play an important role in the pathogenicity of both enterococcal species. This could also be explained that the horizontal virulence gene transfer is common among the clinical isolates.

## Figures and Tables

**Table 1:** Characteristics of different primers used in the current study.

Primer name	Sequence (5′-3′)	Size (bp)	Accession number
*pilA *	Forward: AAAACGCCACCAGAGAAGGT Reverse: CATTGGCGCAATCACAACCA	459	ENA∣ACI49671

*pilB *	Forward: GATACCCAGCTGACGGCTTT Reverse: GGTACTGCCGAAAACGAAGC	959	EFF34880

*fms8 *	Forward: AGACGAGCAGATGAACAGCC Reverse: CCCGTCAATCGTCGTACTGT	765	YP_006376887

*efaAfm*	Forward: AAAAGGCAAGCGACGCAGAT Reverse: AGGTCTAGCCAAGCATGAGG	199	FJ609170

*sgrA *	Forward: CTGATCGGATTGTTTATGA Reverse: AATAAACTTCCCCAATAACTT	150	AGS75503

*ecbA *	Forward: GGAGTGAGGCTTTTAAACCAGA Reverse: GGAAACAGGGTACTTTG	182	AGS75851

*hyl *	Forward: CCCTGGACACATGAAATGCG Reverse: AGCATCGGCCGTTGATAGAC	605	AF544400

*scm *	Forward: GTTTACTAGTCCTAGTTGC Reverse: TCTGTACTGTCGCTTGTGTC	1015	YP_006377279

**Table 2:** Distribution of different virulence factors in *E.  faecium *and *E.  faecalis*.

Virulence genes	*E. faecalis *	*E. faecium *	Total
	(*n* = 50)	(*n* = 29)	(*N* = 79)
*pilB *	50 (100%)	29 (100%)	79 (100%)
*fms8 *	50 (100%)	29 (100%)	79 (100%)
*efaAfm *	50 (100%)	29 (100%)	79 (100%)
*sgrA *	50 (100%)	29 (100%)	79 (100%)
*scm *	44 (88%)	29 (100%)	73 (92.4%)
*ecbA *	35 (70%)	29 (100%)	64 (81%)
*pilA *	29 (58%)	29 (100%)	58 (73.4%)
*hyl *	4 (8%)	24 (82.7%)	28 (35.4%)
